# Prevalence and factors associated with unmet need for family planning among the currently married reproductive age women in Shire-Enda- Slassie, Northern West of Tigray, Ethiopia 2015: a community based cross-sectional study

**DOI:** 10.11604/pamj.2016.23.195.8386

**Published:** 2016-04-15

**Authors:** Gelawdiwos Gebre, Nigussie Birhan, Kahsay Gebreslasie

**Affiliations:** 1 Department of Midwifery, College of Medicine and Health Sciences, University of Mekelle, Ethiopia; 2 Department of Nursing, College of Medicine and Health Science, University of Gondar, Ethiopia; 3 Department of Midwifery, College of Medicine and Health Sciences, University of Gondar, Ethiopia

**Keywords:** Married women, family planning, unmet need, shire, Ethiopia

## Abstract

**Introduction:**

Unmet family planning is one of the common causes for low contraceptive prevalence rate in developing countries including Ethiopia. Thus, this study designed to assess the prevalence and associated factors of unmet need in Shire Endaslassie town, Northern west of Tigray, Ethiopia.

**Methods:**

A community based cross sectional study design was employed. Multistage sampling technique was employed and data were collected using a semi-structured questionnaire by interviewer administered technique. Questionnaires were reviewed and checked for completeness, accuracy and consistency. Reviewed data were entered to Epi info 7 and analyzed by SPSS version 20 statistical software. Variables with P-value of less than 0.2 in bivariate analyses were entered for multivariate analysis and AOR at 95% CI with p-value of less than 0.05 were considered as significant variables.

**Results:**

The overall unmet need for family planning in the study area was 109(21.4%). 74(14.5%) for spacing and 35(6.9%) for limiting. Age group of 35-39 and >=40 (AOR= 2.7,95%CI:1.1,6.5), (AOR = 2.65, 95%CI:1.10, 6.40) respectively, decided numbers of desired children more than five (AOR = O.48, 95%CI: 0.28, 0.80), discussions of client with heath care providers (AOR = 6.32, 95%CI: 2.56, 15.58), previous use of modern family planning (AOR = 2.29, 95%CI, 1.20, 4.34) were significantly associated with unmet need for family planning.

**Conclusion:**

Unmet need for family planning in the study area was high, so continuous discussion on modern family planning with community health workers and encouraging of women to decide desired numbers of children of less than five in general are better to be strengthened.

## Introduction

Family Planning is a principal strategy in controlling population growth and promoting maternal and child health through an adequate spacing of births and avoiding unwanted pregnancy. Contraceptive use has increased markedly in the recent years in most developing countries, due to desire for smaller families; however, millions of women still want to delay or avoid pregnancy but are not using contraception to limit or to spacing their birth [1]. The primary aim of family planning programs is to meet up the demand for contraception and thereby reduce or eliminate unmet need. A well-organized family planning program having a substantial information, education, and communication component can, on average, reduce unmet need by 10% and raise contraceptive use by 22% [2]. "Family Planning: The Changing Path of Unmet Need" [3]. Unmet need for family planning is defined as percentage of all fecund reproductive age women who are married and in consensual union and presumed to be sexually active but are not using any method of contraception, either do not want to have more children, "Limiter" or want to postpone their next birth for at least two years, "Spacer" [4–6]. The absolute number of women with unmet need, however, has increased from 127 million to 142 million, because of the growth of population. Asia accounts for 84 million women having unmet need in 2010, followed by sub-Saharan Africa at 32 million [2].

Sub-Saharan Africa continues in 2010 to be the region with the lowest level of CPR, 24% and highest level of unmet need 25%. Among the 35 countries in sub-Saharan Africa, 24 have unmet need of more than 20 percent, and in 7 of these it was more than 30 percent in 2011 [2]. The percentage of women having unmet need varies broadly across countrieswith the highest levels of unmet need observed in Oceania and Sub-Saharan Africa. The level of unmet need in Latin America and the Caribbean ranges from 9% in Colombia to 35% in Haiti, in Asia ranges from 11% in Indonesia to 32% in Timor-Leste and in Africa, ranges from Egypt (12%) and highest in Sao Tome and Principe (38%) and in Ghana and Liberia (36% each) [7, 8].

Global decreasing of unmet need would prevent around 30% of maternal deaths, reduce child mortality by up to 20%, and avert 36 million women of healthy life lost each year [9, 10]. Helping women and couples plan their families and increased access to contraceptive services to reduce unmet needs would contribute directly to attaining three MDGs: reducing child mortality, improving maternal health and promoting women's empowerment and equality [11]. Reducing unmet need would significantly reduce unintended pregnancy, unsafe abortions, and maternal and child deaths significantly. In Sub-Saharan Africa for instance, it is estimated that provision of family planning services reduces unintended pregnancy by 77% (i.e. from 17 million to 4 million annually), unsafe abortions from 5.2 million to 1.2 million and the number of women in need of medical care from unsafe abortion from 2.2 million to 500,000. So it is wisely clear that family planning is a crucial economic investment [12]. One of the consequences of unmet need is unwanted pregnancy with its serious squeal of induced abortion, and ultimately results in high maternal morbidity and mortality. The risk would increase in developing countries considerably. Thus, meting the unmet need and spacing among births for at least two years are relevant to avoid these deaths and morbidity [13]. Globally about 222 million women have an unmet need for family planning and 645 million women have their needs met through the use of a modern contraceptive method [14]. Among the more than 220 million women with unmet need, three regions-sub-Saharan Africa, South Central Asia and Southeast Asia are dwelling to more than half of these women [3].

Different study shown that, about 8-25% of young women in some Sub-Saharan African countries drop out of school due to unplanned Px as a consequence of unmet needs for FP [15, 16] The 2011 EDHS indicated that Ethiopia with high level of unmet need, that is, 25% of women had unmet need for FP (16% for spacing and 9% for limiting) [17]. Reducing the unmet needs averts unsafe, secret abortion, and its outcomes greatly as the recent reports suggested that only 27% of the 382,000 induced abortions that occurred in 2008 were legal and Some 52,600 women were hospitalized for complications from unsafely induced abortion [18]. A discussion between service provider and client, women with their husband, rural residence and early marriage before 18 years and support has relevant input to meet the unmet needs as per the studies done in, in Kobboworeda, North- East of Amhara and India [19, 20].

## Methods

Community based cross sectional study was conducted among marriage reproductive age in Shire town. The study was conducted in Shire town which is located in 1087km away from Addis Ababa, the capital of Ethiopia. Shire town has a total population of 55, 134, female in reproductive age groups (15-49 years) are 12,456. The study was conduct from March to October, 2014. The study population was consisted of all married women or in consensual union, in the reproductive age group, fecund who live in the selected kebeles of Shire-Enda-Slassie. Currently married reproductive age women who were living with their husband at the period of data collection were included in the study.

Multi-stage sampling technique was used in this study. In the first stage, three out of the five kebeles of the town was selected to represent the urban occupants by using simple random sampling technique/lottery method. Based on the number of reproductive age women in each Kebele, samples was allocated to each Kebele proportionally. In the second stage, in each three kebeles there were four health extension workers dividing each kebeles in to four menders. The lists of identification number as a frame of households were obtained from the health extension workers in each mender. The households in the selected kebeles were randomly selected (reached) by systematic random sampling technique. Using single population proportion formula the finally sample size was found to be 510.

Data was collected by face to face interviews using a structured and pre-tested questionnaire. Training was given for both data collectors and supervisors. Data entry was done by using EPI Info 7 and exported to SPSS version 20 software package for analysis. Multivariate logistic regression was fitted to determine the effect of various factors on the outcome variable. The degree of association between independent and dependent variables were assessed using odds ratio with 95% confidence interval. Ethical clearance was obtained from Institutional Review Board (IRB) of University of Gondar. Formal letter of cooperation was written for Gondar Woreda health department and each health institution. Verbal and written consent was obtained from each study participant.

## Results

### Socio-demographic characteristics

A total of 510 currently married, in consensual union RAW were included in the study with a response rate of 100%. The mean age of respondents was 30.73 (± 6.88 SD) years. Majority 398(78%) of the respondents were followers of Christian orthodox. Most ethnic group of the respondents were, Tigray 479 (93.9%) and regarding to their educational status, majority of them were secondary school completed 231 (45.3%). In relation to occupation, most of the women were house wives 245 (48%). Regarding to monthly income of the respondents, 260 (51%) have monthly income (Table 1).

**Table 1:** Frequency distribution of Socio-demographic characteristics of currently married RAW in Shire Endaslassie town, Northern west of Tigray, Ethiopia, 2015

Characteristics	Frequency	Percent
**Age of respondents**		
15-19	16	3
20-24	92	18
25-29	127	25
30-34	107	21
35-59	98	19
>= 40	70	14
**Religion**		
Orthodox	398	78
Muslim	110	22
Others	2	0.4
**Ethnicity**		
Tigray	479	94
Amhara	22	4
Others	9	2
**Educational Status**		
No formal Education	134	27
Primary Education Completed	53	11
Secondary School Completed	231	54
Higher Level Education	92	18
**Occupation**		
House wife	245	48
Governmental Employee	105	21
Merchant	104	20
Others	56	11
**Monthly Income**		
No	250	49
Yes	260	51
**If yes, how much**		
<600 ETB	38	8
600-1044ETB	98	19
1045-1599ETB	36	7
>1600ETB	88	17

### Reasons for not use of FP methods

From the total study participants, 247(48.4%) were family planning users at the time of data collection, 178 (34.9%) for spacing and 69(13.5%) for limiting. While the currently pregnant and given birth in the last six months with unmet need were asked why not avid it, majority, 46(9%) of them said due to fear of the side effects. On the other hand those not currently pregnant who are not using modern family planning, majority 102(20%) of them responded as they want to have more children followed by 17(3.3%) due to fear of side effects. Over all the main reasons for not to using modern family planning were, Fear of side effects 63(12.4%), Husband disapproval 31(6.1%), little perceived possibility of pregnancy 16(3.1%), followed by religious prohibition15 (2.9%) and fear of infertility 6(1.2%). The CPR of the study population was 247(48.4%) and the unmet need for family planning was109 (21.4%) of them, 14.5% for spacing and 6.9% for limiting. Furthermore, the demand for family planning of the study population was 69.8%.

### Factors associated with Unmet Need for Family Planning

After descriptive analysis was done, bivariate and multivariate logistic regression analysis were carried out. In the analysis of bivariate logistic regression, variables with P-Value of < 0.2 or associated with outcome variable were, age, religion, educational status, occupation, number of live children, decided number of children, the time at which pregnancy can occur while nursing, decision maker on family planning use, discussion with husband about family planning, Partner support for family planning use, discussion with community health providers about family planning and previous use of modern family planning of the respondents. In the multivariate analysis, age group of 35-39 and >=40, religion, decided number of desired children greater than five, discussion with community health providers about family planning, and previous use of modern family planning were significantly associated with unmet need for family planning. As the age increased, the level of unmet need was increased. The married women who were in the age groups of 35-39 and >=40 were positively and significantly associated to unmet need as compared to women in the age group of 15-19 years old (AOR= 2.7, 95%CI: 1.1, 6.5) and (AOR = 2.65, 95%CI:1.10, 6.40) respectively. Women who have decided to have less than five children were 48% less likely to face unmet need for FP over those who decided to have more than five children (AOR = O.48, 95%CI: 0.28, 0.80). Having discussion with community health providers about family planning was negatively and significantly associated to unmet need. Those women who never discussed with the community health providers about family planning were 6.3 times more likely to have unmet need for family planning compared to individuals who had discussed with the providers (AOR = 6.32, 95%CI: 2.56, 15.58). Those women who have never used modern family planning before were 2.29 times more likely to have unmet need for family planning than those who have ever used, therefore, previous exposure to modern family planning has negatively and significantly association to the outcome variable (AOR = 2.29, 95%CI, 1.20, 4.34) (Table 2).

**Table 2:** Logistic regression analysis of associated variables with total of Unmet Need for FP among currently married RAW in Shire Endaslassie town, Northern west of Tigray, Ethiopia, 2015

Independent Variables	Unmet need for family planning		COR(95%CI)	AOR(95%CI)
	Yes	no		
**Age of respondents**				
15-19	1	15	1	1
20-24	17	75	3.4(0.4, 27.5)	0.30(0.03, 2.72)
25-29	25	102	3.67(0.46, 29.16)	1.60(0.61, 4.18)
30-34	25	82	4.57(0.57, 36.35)	1.92(0.79, 4.66)
35-39	25	73	5.13(0.64, 40.89)	**2.71(1.11, 6.64)**^**+**^
>= 40	16	54	4.44(0.54, 36.28)	**2.65(1.10, 6.40)** ^**+**^
**Educational status**				
No formal education	35	99	1	**1**
Primary	13	40	1.67(0.86, 3.25)	
Secondary	45	186	1.54(0.67, 3.52)	
Higher education	16	76	1.14(0.61, 2.15)	
**Occupation**				
House wife	53	192	1	**1**
Governmental employee	19	86	0.80(o.44, 1.43)	
Merchant	28	76	1.33(0.78, 2.26)	
Others	9	47	0.69(0.32, 1.50)	
**Age at first marriage**				
At < 18 years old	60	205	1	**1**
At >=18 years old	49	196	0.85(0.55, 1.30)	
**Desired No of children**				
<5	55	276	0.46(0.30, o.71)	0.48(0.28, 0.80)*
>5	54	12	1	**1**
**Discussion on modern family planning with Community health worker**				
Yes	88	390	1	**1**
No	21	11	8.46(3.93, 18.18)	**6.32(2.56, 15.58)**
**Have you ever used modern family planning**				
Yes	28	361	1	**1**
No	31	40	3.58(2.11, 6.08)	2.29(1.20, 4.34)*

## Discussion

The prevalence rate of unmet need for family planning in this study was 21.4%, for spacing 14.5% and for limiting 6.9% which is in line with Tigray region, 22%(15% for spacing and 7% for limiting, EDHS 2011) and result of Girar Jarso district, north Shoa zone, Oromia national regional state, Ethiopia 2010, 21% that comprising 14% for spacing and 7% limiting [21] and nearly similar with result of research done in Mosul City, North of Iraq 2010, 20.2% [22]. This similarity might be due to comparability in the study design and population, parallel awareness and strategy towards reduction of unmet need.

The result is also in the range with a research results done in Vanuatu and the Solomon Islands, (more than 1000 Islands) 2013, that was, between 11 and 30% have an unmet need for contraception [23]. Might be similar reasons mentioned above. However, it is higher than the results found in the study done, in Uttar Pradesh, India 2012, 13% [3] and UHTC area of Government Medical College Bhavnagar, India 2013 18.7% [24]. This discrepancy might be due to the behavioral, set up and socio-cultural differences and advanced women empowerment on decision making towards fertility goals and preference, awareness on unmet need as well between the studied populations. On the other hand, this level of unmet need is lesser than the results done abroad the country in, urban area of Chidambaram Tamil Nadu, India,2014, 39% (unmet need for spacing was found to be 12 and 27% for limiting of births) [8], Simichaur VDC of Gulmi District of Nepal, 2012, 48%, of them 18.3% were spacers and 29.7% were limiters [25], eastern Sudan, Kassala state,2013, 44.8%, of them 28.1% was for spacing while 16.7% was for limiting [26] and Eritrea,2011, it was 27% with 21% for spacing and 6% for limiting [27]. This discrepancy might be due to the difference in expanded health service provision, initiation and scaling up of health extension workers and consistent implementation of MDG 3 and 5 and some achievements of it, difference of study population, vast investments focus on maternal health by the government.

Discussion between health Service provider and client has relevant input to meet the unmet needs for family planning. Statistically; having discussed with community health providers about family planning was negatively and significantly associated to unmet need. Those women who never discussed with the community health providers about family planning were 6.3 times more likely to have unmet need for family planning when compared to individuals who have discussed with the providers. This is in line with results done in Northwest Ethiopia, Kobboworeda, North- East of Amhara and India [19, 20]. This might be due to similar study design and comparable emphasize given by the local health offices on the outcome of interest. As age of women increased, level of unmet need also increased. The women who were in the age group of 35-39 and >=40 were 2.71 and 2.65 times more likely to have unmet need as compared to women in the age group of 15-19 respectively. But supported by the EMDHS 2014 as Current contraceptive use is lower among currently married women age 40 and above than younger women. 20% for 45-49 versus 40% for less than 40 years of age and Contraceptive use is highest among currently married women age 20-24 (46%) [22]. This might be due to better awareness on unmet need in the lower age groups because of near schooling period in which youth friendly health service, HIV club, reproductive health and rights and other related clubs and services are provided as well as the lower age group are considered to have less number of children that fortune to have time for health service visits and counseling on unmet need and related issues than the upper age groups in the study area.

Previous exposure to modern family planning has negatively and significantly association to unmet need for family planning. Those women who have never used modern family planning before were 2.29 times more likely to have unmet need than those who have ever used. This is supported by the research done in Belesa, north Gondar [28]. This might be from the fact that, ever user are familiar with the service laterally get information from the providers that help them have more awareness and to be less likely to prone for unmet need for family planning.

## Conclusion

Unmet need for FP was found to be high in the study area. Discussions of the women with the community health service providers, previous consumption of FP methods, decided number of desired children more than five and age of women were significantly associated with the outcome variable. FP related discussion of clients is better to accomplished with community health providers to minimize unmet need and pre-pregnancy decision on the desired number of less than five children is very important to have plan on fertility status and goals to decrease unmet need. Continuous health education to bring behavioral change specifically on prohibitive issues to use family planning, miss conception on side effects of family planning and women empowerment to decide number of children and so worth.

### What is known about this topic

- Known factors for unmet need was absence availability of contraception, far from health institution and region

### What this study adds

- Age, decided numbers of desired children more than five, and previous use of modern family planning as factors for unmet need
